# Impact of welfare cheque issue days on a service for those intoxicated in public

**DOI:** 10.1186/1477-7517-4-12

**Published:** 2007-04-26

**Authors:** Xin Li, Huiying Sun, David C Marsh, Aslam H Anis

**Affiliations:** 1 Department of Healthcare and Epidemiology, University of British Columbia, Vancouver, Canada; 2 Centre for Health Evaluation and Outcome Sciences, Vancouver, Canada; 3 Vancouver Coastal Health, Vancouver, Canada; 4 Providence Health Care, Vancouver, Canada

## Abstract

In British Columbia (BC), the Ministry of Human Resources issues welfare cheques to eligible recipients monthly on the last Wednesday of each month. Previous studies have indicated that there are significant increases in hospital admission, ED admission, 911 calls and deaths shortly after the distribution of the monthly welfare cheques. The objective of this analysis was to rigorously examine the impact of welfare cheque issue dates on admission to the Sobering Unit (SU), a service for the publicly intoxicated, in Vancouver, Canada. Data on 1234 consecutive admissions to the SU over a 7-month period were assessed, and the average number of daily admissions on each of the 7 days of the welfare cheque issue week and similar weekdays in other weeks were compared. A Wilcoxon rank-sum test was performed for the comparisons. Our results showed that there were significant increases in the number of admissions on the 3 days starting with "Welfare Wednesday" compared to the similar weekdays in other weeks (Welfare Wednesday vs. other Wednesdays: 8.7 vs. 5.1, p = 0.02; Welfare Thursdays vs. other Thursdays: 9.6 vs. 5.3, p = 0.02; Welfare Fridays vs. other Fridays: 8.6 vs. 5.7, p = 0.04). The demonstrated impact of welfare cheque issue dates is an important consideration for the re-design, staffing and resource allocation of services for withdrawal management and potentially for other services offered to this population.

## Findings

In British Columbia (BC), the Ministry of Human Resources issues welfare cheques to eligible recipients monthly on the last Wednesday of each month. Previous studies in the public health literature, primarily from the United States, have shown that there are significant increases in hospital admission, ED admission, 911 calls and deaths shortly after the distribution of monthly welfare cheques [1-4]. Similar results have also been found in Canadian studies, in which the authors show that welfare cheque issue dates are associated with an increase in the likelihood of an overdose [5], an increase in morbidity and mortality [6], an increase in hospital inpatients leaving a specialized HIV inpatient ward AMA [7] and a decrease in occupancy rate to a medical withdrawal management [8].

This study is designed to rigorously examine the impact of welfare cheque issue dates on admission to the Sobering Unit (SU) in Vancouver, BC. In particular this study will examine how the level of substance use in the community, reflected by the number of public intoxicants brought by the police to the SU, is related to the welfare cheque issue dates. As well the impact of these dates on the utilization patterns of services designed to serve those with substance-related problems will be examined.

The SU is a 15-bed facility operated by Vancouver Coastal Health (VCH) that offers a supportive environment for intoxicated individuals who do not have a safe place in which to recover from the effects of substances. The service includes nursing staff on a 24-hour, 7-day-per-week basis and is designed for those who come to the attention of the police because of substance intoxication in public. It is located in Vancouver's Downtown Eastside (DTES), the most impoverished urban neighborhood in Canada with a high proportion of welfare recipients, and home to people with substance use disorders and injection drug use [9,10]. As a result, the SU service is aimed at the most vulnerable and marginalized individuals who use substances. Because of the target population of the SU, it is anticipated that the majority of those served in this setting will be eligible for social support payments and therefore, this is an ideal setting in which to evaluate the association between welfare cheque issue dates and substance use. Individuals admitted to the SU are brought by the police after being found intoxicated, and often disruptive in the community. It is an alternative to incarceration and clients can be held involuntarily until they are competent to decide on further care. Medications may be administered in the SU to prevent harm to the client or others. If withdrawal care is needed and requested following resolution of intoxication, they are matched to the most appropriate level of care based on client needs.

Our hypothesis is that the level of substance use in the community is driven by the date of welfare payments. Specifically, we hypothesize that the number of admissions to the SU will fall towards the end of the payment period as substance use falls in the community and rise abruptly on the days commencing with "Welfare Wednesday". All these trends would support the concept that the level of substance use in this marginalized population rises and falls in concert with the payment of social benefits.

Therefore, to test the hypothesis, we compared mean daily number of admissions on each of day in welfare weeks with those on similar weekdays in other weeks. Because the data was not normally distributed, a Wilcoxon rank-sum test was performed for the comparisons.

In addition, our analysis will also inform the overall utilization of these services. Any demonstrable impact of welfare cheque issue dates will be important for the design, staffing and resource allocation for these services and potentially for other services offered to this population.

The present study extracted seven-month data (August 1, 2003 to Feb. 29, 2004) from VCHs Primary Access Regional Information System (PARIS) database. Ethical Approval for the study was obtained from the Behavioural Research Ethics Board of the University of British Columbia.

**Figure 1:**
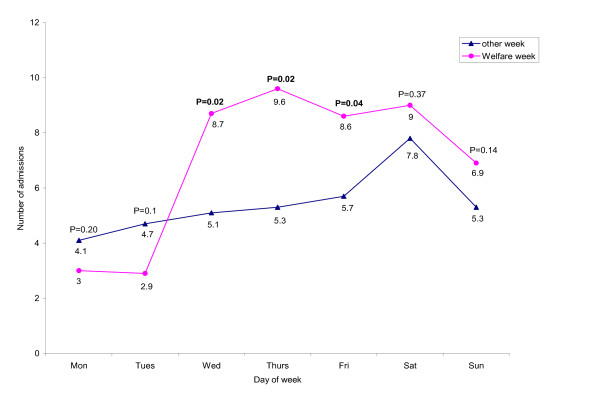
Comparison of the mean number of SU admissions on a given day of the welfare week and the mean daily admission number in all other weeks. P = P value.

Overall, 1234 admissions were made to the SU during the study period, the median age was 40 (Q1–Q3: 30–49), and the majority was male (80%). Most of clients, 854 (69%), were discharged from the SU at the same day of admission, 366 (30%), stayed overnight, and 14 (1%) stayed more than one night. Figure 1 illustrated the mean number of SU admissions on specific weekdays in the welfare weeks and the mean daily admission number in all other weeks. The average daily admission peaked on Saturdays in other weeks. This might be due to an increase in substance use over the weekends. We did not find significant changes in the number of admissions on Mondays and Tuesdays in welfare weeks compared to those on other Mondays and Tuesdays, although the numbers in welfare weeks were lower (Mondays: 3.0 (95% CI = 1.07–4.93) vs. 4.1 (95% CI = 3.37–4.89), P = 0.20; Tuesdays: 2.9 (95% CI = 0.83–4.89) vs. 4.7 95% CI = 3.54–5.94), P = 0.14). On the other hand, we found a significant increase in the number of admissions on each of the 3 days commencing with "Welfare Wednesday" compared to the similar weekdays in other weeks. Specifically, the number of admissions on "Welfare Wednesday" was 71% higher than that on other Wednesdays (8.7 (95% CI = 5.71–11.72) vs. 5.1(95% CI = 4.24–5.93), P = 0.02), and 81% and 51% higher on Thursdays and Fridays following "Welfare Wednesday" compared to other Thursdays and Fridays, respectively (Thursdays: 9.6 (95% CI = 6.33–12.81) vs. 5.3 (95% CI = 3.87–6.65), P = 0.02; Fridays: 8.6 (95% CI = 6.13–11.01) vs. 5.7 (95% CI = 4.45–6.96), P = 0.04). In terms of weekend comparisons, although we found the number of admissions during welfare weekends were higher than those in other weekends, the differences were not statistically significant (Saturdays: 9.0 (95% CI = 6.03–11.97) vs. 7.8 (95% CI = 6.1–9.48), P = 0.37; Sundays: 6.9 (95% CI = 4.83–8.89) vs. 5.3 (95% CI = 4.26–6.41), P = 0.14).

Our study demonstrates a clear temporal relationship of increased medical events to "Welfare Wednesday" by showing that there are significant increases in the number of admissions to the SU following "Welfare Wednesday". The findings indicate that in addition to buying food, or paying rent, some welfare recipients may use the money to buy substances. Simply discontinuing the welfare payments to substance users is unlikely to be an attractive policy option since it will not eliminate substance use and might exacerbate hunger and homelessness [11]. One possible solution is to appoint a payee who receives and manages welfare income on behalf of the substance users. Specifically, the payee could dispense the money through the course of the month or arrange for its use for food and shelter. However, the effectiveness of payees approach is still under debate [11-13]. Another controversial proposal is to require addiction treatment as a condition of receiving welfare. Specifically, those with positive tests could be required to receive treatment and abstain from substance use or risk losing their benefits. However, this proposal is not supported by evidence and may produce increases in crime, health problems and other social costs [14].

Our results also highlight the importance of interventions in preventing the potential variability in demand for the SU due to the dates of welfare payments. The decreased admissions right before "Welfare Wednesday" and the increased admissions starting from "Welfare Wednesday" indicate that the demand for the SU service does not smooth out over a month but is peaked in the "Welfare Wednesday" weeks. This sudden increased demand for the SU service could present pressures on the system including the police and health care resources. Two possible interventions might be used to solve the uneven demand problem. The first intervention could be done by health care providers, in which staffing and resource allocation for the services could be based on the demand for the service. Specifically, as demand goes up, more staff and more health care resources should be allocated. The reverse could happen when demand goes down. The second possible intervention could be done by policy makers of the provincial government, in which distribution of welfare cheques could be spread out over a month. For instance, cheques could be distributed on the individual's birthday. Doing so could spread out the demand for the SU service, therefore eliminating the variability in demand. This could also decrease the negative impact on other health care resources resulting from the peak in community substance use due to the current pattern of welfare payment.

Our study has several limitations. First, our study cannot isolate other forms of payment from welfare payments since we could not isolate clients on assistance. However, it is likely that the effect will be more dramatically shown if only the clients receiving welfare were tracked rather than using the whole sample. Second, our study was conducted at a single sobering unit. Thus, our results may not be generalizable to patterns in other settings. However, previous studies have shown that the timing of welfare cheque issue is associated with an impact on utilization of other health care services.

In summary, fewer admissions are made to the SU right before "Welfare Wednesday", and more admissions are made on each of the 3 days commencing with "Welfare Wednesday". The demonstrable impact of welfare cheque issue dates will be important for the design, staffing and resource allocation for these services and potentially for other services offered to this population.

## Competing interests

The author(s) declare that they have no competing interests.

## Authors' contributions

All coauthors made significant contributions to the conception and design of the analyses, interpretation of the data and drafting of the manuscript, and they all approved the version to be published.
